# Comparison of Bacterial Assemblages Associated with Harmful Cyanobacteria under Different Light Conditions

**DOI:** 10.3390/microorganisms10112150

**Published:** 2022-10-30

**Authors:** Taehui Yang, Chang Soo Lee, Ja-Young Cho, Mi-Jung Bae, Eui-Jin Kim

**Affiliations:** 1 Gossong Deep Sea Water Industry Foundation, Gossong-gun 24747, Korea; 2 Nakdonggang National Institute of Biological Resource (NNIBR), Sangju-si 37242, Korea

**Keywords:** cyanobacteria, light limitation, *Anabaena* sp., *Microcystis* sp., cyanobacteria-associated bacteria, non-metric multidimensional scaling

## Abstract

Harmful cyanobacterial blooms in freshwater ecosystems are closely associated with changes in the composition of symbiotic microbiomes, water quality, and environmental factors. In this work, the relationship between two representative harmful cyanobacterial species (*Anabaena* sp. and *Microcystis* sp.) and their associated bacterial assemblages were investigated using a 16S rRNA-based meta-amplicon sequencing analysis during a large-scale cultivation of cyanobacteria under different light conditions with limited wavelength ranges (natural light, blue-filtered light, green-filtered light, and dark conditions). During the cultivation periods, the growth pattern of cyanobacteria and bacterial composition of the phycosphere considerably varied in relation to light restrictions. Unlike other conditions, the cyanobacterial species exhibited significant growth during the cultivation period under both the natural and the blue light conditions. Analyses of the nitrogenous substances revealed that nitrogen assimilation by nitrate reductase for the growth of cyanobacteria occurred primarily under natural light conditions, whereas nitrogenase in symbiotic bacteria could also be activated under blue light conditions. *Sphingobium* sp., associated with nitrogen assimilation via nitrogenase, was particularly dominant when the cell density of *Microcystis* sp. increased under the blue light conditions. Thus, cyanobacteria could have symbiotic relationships with ammonium-assimilating bacteria under light-limited conditions, which aids the growth of cyanobacteria.

## 1. Introduction

Cyanobacterial blooms are detrimental to human health and freshwater ecosystems [1]. *Anabaena variabilis* (syn. *Trichormus variabilis*) and *Microcystis aeruginosa* are representative and ubiquitous freshwater cyanobacteria that have garnered much attention over the past decades owing to the water management strategies necessitated to mitigate the effects of their blooms. To regulate harmful cyanobacterial blooms (HCBs), various factors, such as the bacterial community composition associated with cyanobacteria, nitrogenous and phosphorus compounds, water quality, temperature, and light conditions, should be systematically considered. Among the various factors, light is one of the major limiting factors regulating cyanobacterial growth [2]. Cyanobacteria contain chlorophylls and carotenoids, which can absorb natural light at the corresponding wavelengths for photosynthesis, and the chlorophyll-to-carotenoid ratio can be affected by abiotic (e.g., temperature, light, pH, and salinity) and/or biotic (i.e., pathogen contamination and competition with other microorganisms) stressors [3]. Therefore, the growth pattern of cyanobacteria can be differentiated based on different wavelength ranges of light irradiation [4,5].

The interaction between cyanobacteria and their associated bacteria is another major factor that regulates HCBs in addition to light limitation [6,7]. The composition of cyanobacteria-associated bacteria in the phycosphere varies depending on the species of cyanobacteria and the environmental circumstances during the HCBs [8,9]. Moreover, the growth pattern and the physiological response of cyanobacteria can be severely influenced by associated bacterial species [10]. For example, *Rhizobium* sp. protects the cell wall of cyanobacteria from oxidative stress [10] and secretes algaecide enzymes that indirectly attack the microalgae [11,12,13]. These antagonistic relationships are accompanied by cyanobacterial blooms or declines. Additionally, the functional position of bacteria consisting of a phycosphere can be changed according to the type of symbiotic cyanobacteria. Cyanobacteria and their associated bacteria influence each other through the transport of organic and inorganic compounds. Through nitrogen fixation and organic compound decomposition, bacteria can supply inorganic C, N, P, and other nutrients to the associated cyanobacteria [14]. Moreover, cyanobacteria act as a source of organic and inorganic compounds including proteins, high-molecular-weight carbohydrates, and oxygen, which are required for bacterial metabolism [15].

To interpret the mechanisms of HCBs, finding out the relationships among cyanobacteria, light conditions, and cyanobacteria-associated bacteria is essential. Therefore, we hypothesized that cyanobacteria would adopt a selective strategy to conduct efficient photosynthesis within a specific wavelength range (i.e., under light-limited conditions). In addition, it is assumed that some cyanobacterial taxa have symbiotic relationships with their associated bacteria (i.e., the cyanobacterial phycosphere) owing to the lack of complete metabolic pathways required for the phototrophic growth of cyanobacteria. To mimic the growth patterns and adaptations of cyanobacterial species contributing to HCBs in freshwater ecosystems, two kinds of representative cyanobacteria were simultaneously cultured under light-limited conditions in this study. To verify the hypothesis, we investigated the effects of natural light limitations (using blue and/or green light-penetrating films) on the growth of *Anabaena* sp. and *Microcystis* sp. as well as the changes in the bacterial assemblages of the phycosphere.

## 2. Materials and Methods

### 2.1. Cyanobacterial Strains and Seed Cultivation

*Anabaena* sp. (FBCC-A1343) and *Microcystis* sp. (FBCC-A141) were obtained from the Freshwater Bioresources Culture Collection (FBCC) of Nakdonggang National Institute of Biological Resources (NNIBR, Sangju-si, Korea). The cyanobacterial cell cultures including their associated bacteria (i.e., the cyanobacterial phycosphere) were maintained in BG-11 medium at 20 °C under laboratory conditions with 50 µmol m^−2^ s^−1^ of light intensity using fluorescent light and a 14/10 h light-dark cycle [16,17].

### 2.2. Experimental Conditions for Light-Limited Cultivations

**Figure 1:**
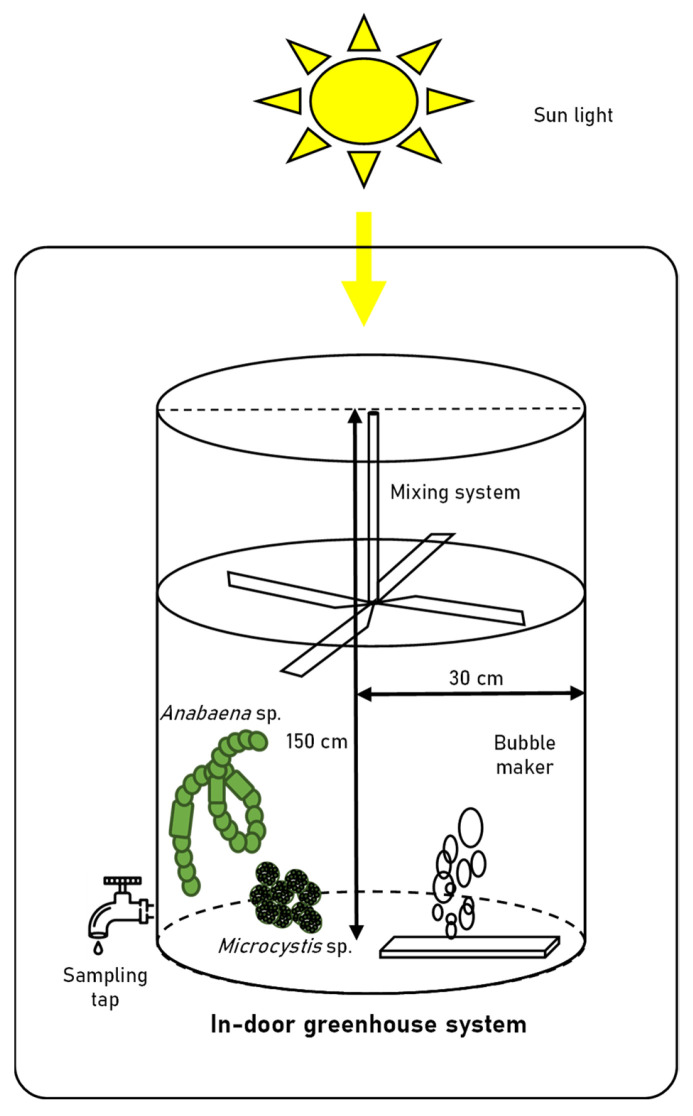
Schematic diagram of large-scale cultivation system.

*Anabaena* sp. and *Microcystis* sp. were cultivated in a 60 cm (diameter) × 150 cm transparent acrylic tank containing 100 L of BG-11 medium (diluted 5-fold) in an indoor greenhouse. The nitrogen source comprised 300 mg/L of NaNO_3_ and 1.2 mg/L of Ferric ammonium citrate in BG-11 medium. The acrylic tanks were covered tightly with a blue-light filter and/or green-light filter (Figure 1). Acrylic tanks were composed of a paddlewheel system to prevent cell settling, and an air sparger (1000 cc/min). The initial seed cultures of *Anabaena* sp. and *Microcystis* sp. were grown in a 2-L mini-column until the cell density of both strains had reached approximately 1.0 × 10^5^ cells/mL and were subsequently inoculated into four different light conditions. The composition of cyanobacteria and their associated bacterial assemblages in the tanks were observed for 15 days. The environmental parameters expected to affect the microbial communities were measured during the experimental period. The spectral composition of natural light was monitored using a portable spectrometer (LI-180, LI-COR, Lincoln, NE, USA) in 1 nm wavelength intervals with a 12 nm bandwidth thrice a day (9 am, 1 pm, and 5 pm) for 15 days. The water temperature, dissolved oxygen (DO), conductivity, pH, and turbidity were recorded during the experimental period using a ProDSS Multiparameter Water Quality Meter (YSI Inc., Yellow Springs, OH, USA). The total nitrogen (TN), total phosphorus (TP), biochemical oxygen demand (BOD), chemical oxygen demand (COD), nitrite (NO_2_^−^), nitrate (NO_3_^−^), ammonia (NH_3_), and orthophosphate (PO_4_^3−^) levels were measured according to standard procedures [18].

### 2.3. Flow Cytometric Analysis for Live-Cell Density Measurement

Cyanobacterial cells were collected from the four acrylic tanks and counted using flow cytometry [19]. A 200 µL sample of culture medium was aliquoted and then stained using fluorescein diacetate (Sigma-Aldrich, final concentration of 2 μM) for 10 min in the dark conditions [20]. A Guava^®^ easyCyte™ flow cytometer (Luminex Corporation, Austin, TX, USA) was used with an excitation blue (488 nm) laser. The forward scatter, side scatter, green fluorescence, yellow fluorescence, and red B fluorescence gain controls were set as 1.30, 1.00, 1.54, 1.61, and 1.30, respectively. The experiment was carried out in triplicate. The aliquoted samples (200 μL) were analyzed using 96-well flat-bottom plates (Corning Life Sciences, Tewksbury, MA, USA) with automatic mixing of each well for 5 s at a high speed before sampling. To analyze the state of cells, a cluster of *Anabaena* sp. and *Microcystis* sp. was each selected to plot the coordination (forward scatter and side scatter) of the cell size of cyanobacteria. Concerning the emission of chlorophylls (red fluorescence range), a cluster of *Anabaena* sp. and *Microcystis* sp. each was again selected for forward scatter and red fluorescence coordination. The selected cyanobacterial clusters were counted based on fluorescence intensity upon plotting coordination (green fluorescence and forward scatter). The number of live cells were calculated automatically based on their morphological and fluorescence characteristics using the flow cytometric analyses by Guava software (Figures S1 and S2). During each sampling step, flow cytometric analysis of *Anabaena* sp. and *Microcystis* sp. cells was performed using all samples in triplicate.

### 2.4. DNA Extraction and Quantification

The culture medium (2L) samples corresponding to every sampling day were used for analyzing the composition of bacterial communities. The bacterial cells in cyanobacterial phycosphere were concentrated using a membrane filter (ADVANTEC) and stored at −20 °C until the DNA extraction step. Genomic DNA (gDNA) of concentrated bacterial samples was extracted with a DNeasy PowerSoil Kit (Qiagen, Hilden, Germany). The extracted gDNA was then quantified using a Quant-IT PicoGreen (Invitrogen).

### 2.5. Preparation of Libraries for Sequencing Analysis

Sequencing libraries were prepared based on the Illumina protocols to amplify the V3 and V4 regions of the 16S rRNA gene. The input gDNA (2 ng) was PCR-amplified using Herculase II fusion DNA polymerase (Agilent Technologies, Santa Clara County, CA, USA) and universal F/R primers. The cycle conditions for the first PCR were 3 min heat activation at 95 °C; 25 cycles of 30 s at 95 °C, 30 s at 55 °C, and 30 s at 72 °C; and a final extension at 72 °C for 5 min. The universal primer pair with the Illumina adapter overhang sequences used for the first PCR were as follows: V3-F:5′-TCGTCGGCAGCGTCAGATGTGTATAAGAGACAGCCTACGGGNGGCWGCAG-3′ and V4-R:5′-GTCTCGTGGGCTCGGAGATGTGTATAAGAGACAGGACTACHVGGTATCTAATCC-3′. The first PCR product was purified using AMPure beads (Beckman Coulter Life Sciences, Indianapolis, IN, USA). Aliquot of (2 µL) the first PCR products were then PCR-amplified for generating the final library containing the index by using the Nextera XT Indexed Primer. The conditions for the second PCR were identical to those used for the first PCR, except that it was performed for only 10 cycles. The PCR product was purified again using the AMPure beads and quantified by qPCR according to the qPCR Quantification Protocol Guide (KAPA Library Quantification kits for Illumina Sequencing platforms). The quality was analyzed with a Tape Station D1000 Screen Tape (Agilent Technologies). The paired-end (2 × 300 bp) sequencing was conducted via Macrogen (Seoul, South Korea) using a MiSeq platform (Illumina, San Diego, CA, USA). The resulting sequence data were analyzed using QIIME and the UPARSE pipeline [21]. The Illumina processing in QIIME was performed using the default settings, and the UPARSE pipeline was applied to assign the taxonomic groups at 97% similarity. Following operational taxonomic units (OTUs) abundance data were normalized.

### 2.6. Data Analysis

We analyzed the data in four steps to interpret the differences in microbial communities among the four experimental conditions. First, alpha diversity [22] was calculated using the QIIME software package. To compare the four experimental conditions, we measured the relative abundance of the bacterial community rarefied the OTU table microbial richness (Chao1) and Shannon’s index using the Mothur software [23]. Second, we classified the sampling sites using a hierarchical cluster analysis (CA) based on the bacterial community. The Bray–Curtis distance and Ward’s linkage methods [24] were applied to calculate the CA. Third, to pattern the differences in the bacterial community according to the light conditions, we used non-metric multidimensional scaling (NMDS) based on the Bray–Curtis distance between sampling sites. We used ‘metaMDS’ to detect the lowest STRESS value and ‘envfit’ function to determine the relationship between the bacterial community and the water quality parameters [25]. The CA and the NMDS analyses were conducted using the ‘vegan’ package in R [26]. Prior to the CA and the NMDS analyses, we removed rare species (i.e., <0.01% relative abundance) and arc-transformed the community data. Finally, we analyzed indicator species to identify representative species from the CA groups. According to Dufrêne and Legendre (1997), the indicator species for each group were selected when the indicator value (IndVal) was significantly higher than 25% (*p* < 0.05). We used the “indval” function to analyze indicator species using ‘Labdsv’ package in R [27].

## 3. Results

### 3.1. Patterns of Cell Growth of Cyanobacteria

**Figure 2:**
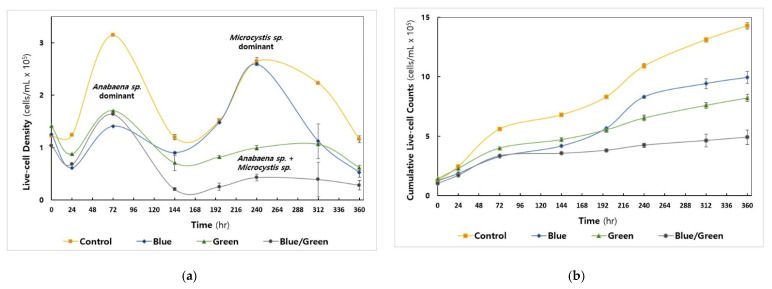
Cyanobacterial cell growth under the experimental conditions (Control: No intervention, Blue: Blue film, Green: Green film, Blue/Green: Blue and Green films). All experiments were carried out in triplicate. (**a**) Measurement of cyanobacterial live-cell density by flow cytometry (**b**) The cumulative number of cells counted.

Among the cyanobacteria, *Anabaena* sp. showed the maximal cell density at 72 h under all conditions examined (Figure 2a), whereas the growth of *Microcystis* sp. was predominantly observed in the control (i.e., natural light condition) and blue (i.e., blue-light filtered condition) conditions in the second live-cell increment phase at 240 h (Day 10, Figure S2f). The control condition reached 3.1 × 10^6^ cells/mL, whereas the other conditions exhibited live-cell density values below 1.5 × 10^6^ cells/mL at 72 h. The total cumulative live-cell density in each cultivation was highest in the control condition (1.43 × 10^7^ cells/mL), followed by the blue, green, and dark conditions (4.9 × 10^6^ cells/mL) (Figure 2b).

### 3.2. Variation in Physico-Chemical Factors during Cultivations

**Figure 3:**
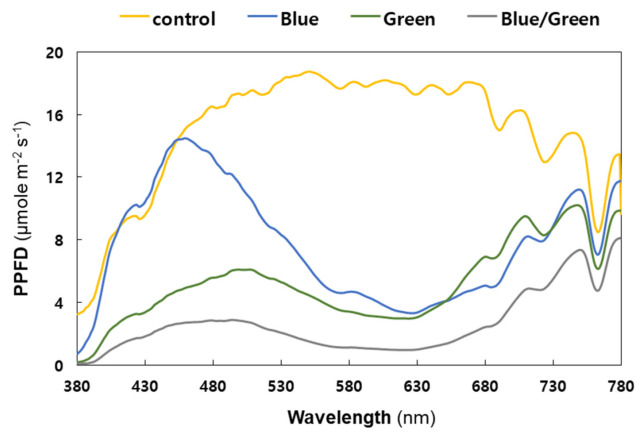
Spectral composition of light under the experimental conditions (Yellow: control, Blue: blue film, Green: green film, Grey: blue and green films). X-axis: wavelength; y-axis: photosynthetic photon flux density (PPFD). The PPFD units were measured in 1 nm wavelength intervals with a 12 nm bandwidth. Each measurement was taken thrice and the values were then averaged.

Figure 3 shows the spectral profile of light intensity measured under four different growth conditions. The control condition absorbed a broad range of light wavelengths, from 380 to 780 nm, with photosynthetic photon flux density (PPFD) above 10 μmol m^−2^ s^−1^ (from 395 to 758 nm), showing the highest cell growth in the first and second increments of cyanobacterial cell density (Figure 2a). The light intensity in the blue condition exceeded 5 μmol m^−2^ s^−1^ in the ranges of approximately 399–562 nm and 678–780 nm only; however, it showed the highest PPFD unit (14.5 μmol m^−2^ s^−1^) measured at 459 nm with a 12 nm bandwidth. Considering that the light intensity in blue-light wavelength range (415−495 nm) was comparable to natural light, the cyanobacterial live-cell growth under the blue condition was similar to that under the control condition during the second increment of cell density around 240 h (Figure 2a).

The water temperature in the cell cultivation systems increased from 24 to 30 °C, and then decreased to approximately 27 °C (Table S1). The DO (%) was the highest at 72 h under all conditions, and the DO value was highest in the control condition (127.3%), followed by the blue (115.2%), green (107.2%), and dark (103.1 %) conditions (Table S1). The pH increased after 24 h, except for the dark condition; notably, the control condition showed the highest pH value at 72 h. The pH value under dark conditions did not change abruptly during the experimental period (Table S1). Turbidity, BOD, and COD increased from 144 h, especially in the control condition (Table 1 and Table S1). Regarding the variation in nitrogenous compounds, nitrate and nitrite concentrations of the control group tended to increase from 144 h to 196 h but decreased immediately from 196 h to 240 h (Table 1), whereas other light-filtered conditions showed different patterns. The ammonia values of the blue condition, which rapidly increased at 196 h and decreased at 240 h, were distinguished from those of the other conditions (Table 1). The values of the phosphorus compounds were reduced throughout the cultivation period under all conditions (Table 1).

### 3.3. Changes in the Bacterial Community Composition during Cultivations

A total of 1,801,250 qualified sequences were obtained after processing, and 414 OTUs were identified at a 97% similarity cut off. These OTUs belonged to 12 phyla, 27 classes, 59 orders, 100 families, and 190 genera. The analysis of alpha diversity analysis (e.g., Chao1, and Shannon index) of each experimental group was carried out (Table S2). The Shannon index of the control and green conditions increased during cultivation, and those of the blue and dark conditions decreased. The Chao1 index of all the experimental conditions showed an increasing trend during cultivation. The rarefaction curves indicated that the phylotype richness in sequenced samples increased during the cultivation period (Figure S3), showing that bacterial communities at the late stage of cultivation were more abundant when compared to those in the early stage.

**Figure 4:**
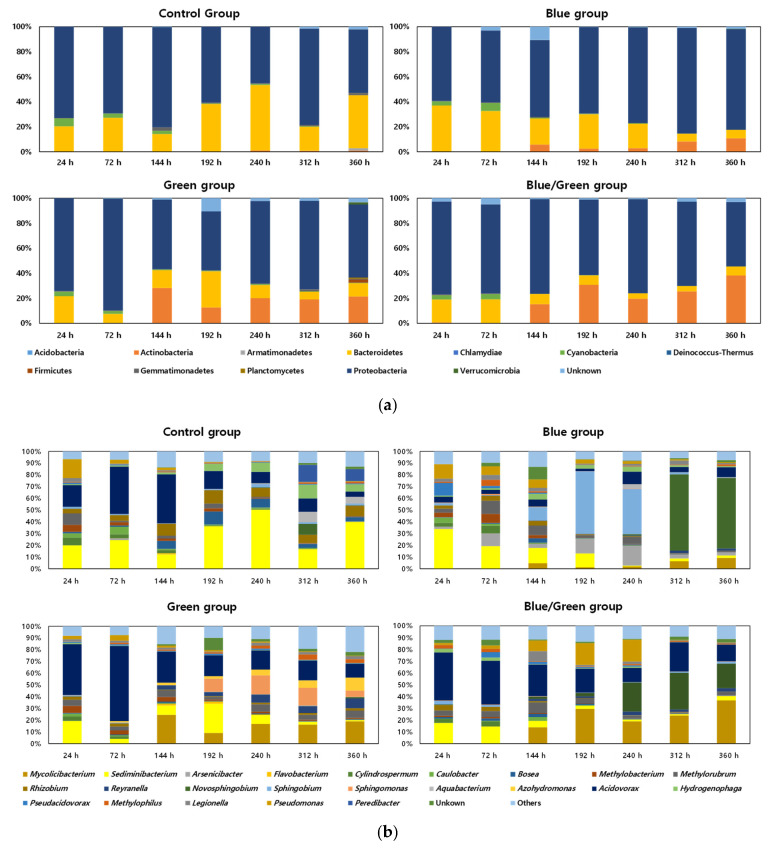
Relative abundance of the bacterial community (x-axis: Cultivation period, y-axis: Relative abundance of bacterial taxa). (**a**) Phylum level; (**b**) Genus level (genera with >5% abundance is listed while the rest are categorized under ‘others’).

The relative abundances of bacterial communities under the four experimental conditions are shown in Figure 4. Proteobacteria and Bacteroidetes were the major phyla in the control condition, whereas Proteobacteria, Bacteroidetes, and Actinobacteria were the major phyla under the other conditions (Figure 4a). The phylum Actinomycetes was found to increase at 144 h under the three light-limited conditions, and the ratio of Actinomycetes in the dark condition reached 38% at 360 h. Moreover, *Acidovorax* and *Sediminibacterium* were the dominant genera in the control condition and showed a higher ratio at 144 h (41%) and 192 h (50%), respectively (Figure 4b). *Sphingobium* and *Novosphingobium* were the dominant genera in the blue condition at 192 h (53%) and 312 h (65%), respectively.

### 3.4. Analyses for Bacterial Assemblages Associated with Harmful Cyanobacteria

**Figure 5:**
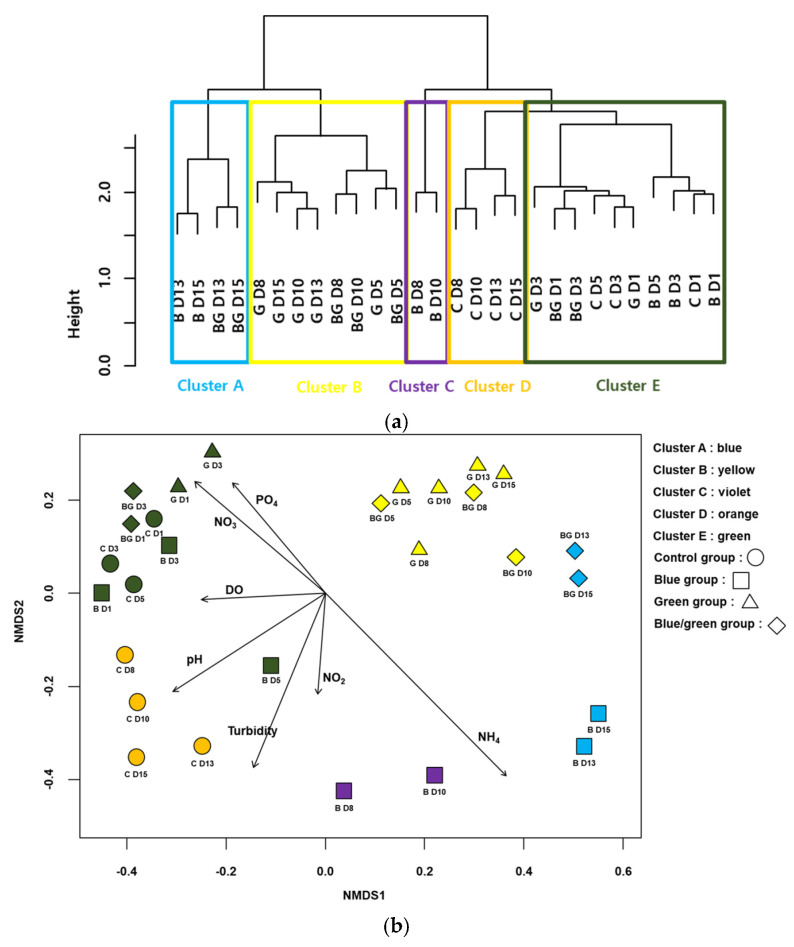
Cluster analysis based on cyanobacteria-associated bacteria assemblages (**a**) Non-metric multidimensional scaling (NMDS) ordination of samplings with cyanobacteria-associated bacteria assemblages (**b**). Arrows indicate the water quality variables significantly related to the assemblage compositions (*p* < 0.05). The longer the arrow length, the higher the correlation coefficients with the axis of NMDS. The first letter in figures indicates the experimental condition (i.e., C: control, B: blue film, G: green film, BC: blue and green light) and the number indicates the experimental date (D1: 24 h, D3: 72 h, D5: 14 h, D8: 196 h, D10: 240 h, D13: 312 h and D15: 360 h).

The CA classified the samples into five clusters (A–E) based on the similarities in the bacterial communities (Figure 5a). All samples from the control condition and the first two samples from the blue, green, and dark conditions were included in cluster E. All samples except the first two samples from the green condition were in cluster B. The last two samples from the blue and dark conditions were in cluster A. The NMDS also represented the differences in the bacterial communities and clusters from the CA (STRESS value = 0.072 for the first three axes) (Figure 5b). Samples with high values of NH_3_ were located on the lower-right side of the NMDS plot, whereas those with high values of NO_3_^−^ and PO_4_^3−^ were located on the upper-left side of the NMDS plot. In addition, samples that showed lower growth of cyanobacteria were primarily located on the upper-right side of the NMDS plot (e.g., the samples from the dark condition).

Fifty-two taxa were selected as indicator species (or taxa) for the five groups from the CA based on IndVal (*p* < 0.05) (Table 2). The number of indicator species was the highest in group D (20 taxa), followed by groups A (11 taxa), E (11 taxa), B (7 taxa), and C (3 taxa). *Rhodobacter* (IndVal = 0.97) was selected as an indicator genus, which includes a group of purple non-sulfur bacteria that can obtain energy through anoxygenic photosynthesis [28] and can also fix molecular nitrogen [29]. Group B consisted of seven taxa, including *Sphingomonas* (IndVal = 0.91), a chemoheterotrophic bacterial genus belonging to the α-proteobacteria and possessing ubiquinone 10 as a respiratory quinone and contains glycosphingolipids in place of lipopolysaccharides [30]. A representative genus in Group C, *Sphingobium* (IndVal = 0.90), is different from other sphingomonads, which can degrade a variety of chemicals (e.g., aromatic and chloroaromatic compounds) in the environment [31]. The genus *Bryobacter* (IndVal = 0.97) belonging to the phylum Acidobacteria is one of the representative genera in Group D and accommodates strictly aerobic acidotolerant bacteria that commonly inhabit acidic environments [32]. *Cupriavidus* (IndVal = 0.97) in group E, which is an obligate aerobic organism that grows chemoorganotrophically or chemolithotrophically, is selected as an indicator genus [33].

## 4. Discussion

Among the four experimental conditions examined, only the natural light condition (control group) exhibited considerable cell growth (3.1 × 10^5^ cells/mL) during the first growth phase of cyanobacteria (from 0 h to 144 h), which was primarily due to the proliferation of *Anabaena* sp. as determined by the flow cytometry. However, the second growth phase (from 144 h to 360 h) was observed in the control as well as the blue-light filtered conditions via the cell proliferation of *Microcystis* sp., indicating that the dominant cyanobacterial genus in the cultivation system can be changed by varying the physico-chemical factors. Our results showed that the culture conditions at the starting point of cultivation were more appropriate for the growth of *Anabaena* sp. compared to *Microcystis* sp. Indeed, *Anabaena* sp. utilized natural light and initially provided nitrate for their growth, which led to a decrease in TN and TP sources at 144 h. However, after the increase in the ammonia concentration at 196 h, possibly generated by some of cyanobacteria-associated bacterial species under the blue light, *Microcystis* sp. was the dominant genera of cyanobacteria.

During cyanobacterial cultivations, the composition of the bacterial communities were significantly influenced by the irradiated wavelength range. In this study, *Acidovorax* sp. dominantly appeared at the first increment of cyanobacterial cell density, except under the blue conditions. These aerobic chemoheterotrophic bacteria, belonging to *β*-proteobacteria, are able to dissimilate organic compounds with nitrate denitrification-related genes [34]. Thus, *Acidovorax* sp. can act as a decomposer in the cyanobacterial phycosphere by utilizing the organic polymers produced by cyanobacteria. *Sediminibacterium* sp. was dominant in the control condition and the variation in their occupying ratio was similar to the growth pattern of cyanobacteria in the period of the second cyanobacterial growth. It was established that *Sediminibacterium* sp. forms biofilm consortia with cyanobacteria [35], and its relationship with cyanobacteria in wastewater has also been previously reported [36]. Thus, *Sediminibacterium* sp. also can be considered to play a role as a supplier of inorganic nutrients for cyanobacterial growth under control conditions.

Two types of ammonia assimilation mechanisms are accomplished by nitrate reductase and nitrogenase. These nitrogen fixation processes can be performed by cyanobacteria themselves and/or bacterial assemblages in a phycosphere. Assimilatory nitrate reduction in cyanobacteria primarily occurs via nitrate reductase and nitrite reductase, which incorporates nitrate into amino acids and amides [37,38] for the constructive production of cells. The changes in the concentrations of nitrogenous compounds (i.e., TN, nitrate, and nitrite) under the control condition suggest that the cell growth of cyanobacteria under natural light systemically depends on the assimilatory nitrate reduction [39,40].

As mentioned above, several studies have reported that light limitations can affect cyanobacterial growth [41,42] as well as the production of their secondary metabolites [43,44,45]. Especially, the irradiation of blue light between 450 nm and 500 nm, which could enhance the production of phycobiliproteins such as phycocyanin, allophycocyanin, and phycoerythrin in cyanobacteria [46,47]. Some cyanobacteria have a long-term acclimation mechanism (i.e., chromatic acclimation) to different light ranges by inducing the synthesis of distinct pigments [48]. During the early stage of the experiment, cyanobacteria under the blue condition probably synthesized carotenoids involved in the phycobilisome protein complex as the light acceptor anchored on the thylakoid for efficient utilization of the low intensity of blue light (400–500 nm). Numerous studies have reported that the production of phycobilisome proteins could be enhanced by a nitrogen source supply in microalgal cultivation [49,50] and increased by the incidence of low-intensity light [51,52].

**Figure 6:**
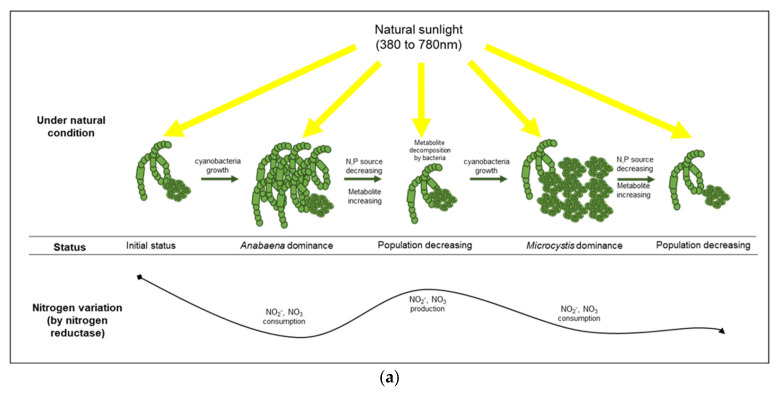

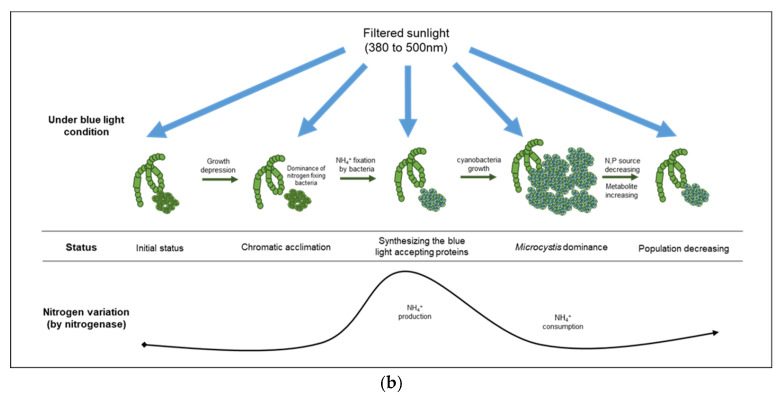
Comparison of cyanobacterial growth patterns under natural (**a**) and blue light-filtered (**b**) conditions. It was elucidated that a plausible interaction exists between cyanobacteria and their associated bacterial assemblages through nitrogenous metabolites.

Under the blue condition, marked changes in the levels of nitrate and nitrite were not observed until the second cyanobacterial growth, whereas a large variation in the ammonia level was observed based on the CA and the NMDS analyses. Therefore, the ammonia produced in the blue condition could be assimilated by nitrogenase originating from cyanobacteria-associated bacteria in the phycosphere. Nitrogenase, an enzyme that directly converts atmospheric nitrogen to ammonia, can exist in several symbiotic bacteria that interact with photosynthetic organisms [53]. Owing to the lack of genes for nitrogenase in *Microcystis* sp., it is likely that the nitrogen-fixing function of a cyanobacterial phycosphere could be complemented by cyanobacteria-associated bacterial community [54,55,56]. The density of *Sphingobium* sp., which belongs to the group of *β*-proteobacteria and is associated with ammonia production [57], increased rapidly (192 h, 53%) before the second cyanobacterial growth, especially in the blue condition. *Sphingobium* sp. has the ability to ammonify NO_2_^−^ to NH_3_ (https://www.genome.jp/kegg-bin/show_pathway?spht00910, accessed on 3 November 2021) and is related to aerobic nitrogen fixation (N_2_ to NH_3_) [58], which indicated that the rapid increase in ammonia at 196 h in the blue condition was primarily due to the abundance of *Sphingobium* sp., which led to the cyanobacterial growth by *Microcystis* sp. at 240 h in the blue condition (Figure 6).

On the other hand, *Mycolicibacterium* sp., belonging to the phylum *Actinobacteria*, appeared primarily in the middle and late stages of the cultivation period under both the green and dark conditions. *Mycolicibacterium* sp. have been reported to be pathogenic [59,60,61] and can degrade non-biodegradable compounds, such as polyphenyl and polyhydrocarbon. Thus, *Mycolicibacterium* sp. are probably involved in decomposing high-molecular-weight organic compounds produced by other bacteria in low light intensity under both green and dark conditions.

This study has certain limitations and can be further augmented on two counts: (1) maintaining the cultivation temperature constantly, and (2) separately calculating the cell numbers of *Anabaena* and *Microcystis*. First, even though our experimental temperature of the large cultivation system was not maintained constantly, the temperature variation from 25 °C to 30 °C may be suitable for cyanobacterial cultures [62]. The formation of cyanobacterial blooms containing *Anabaena* sp. and *Microcystis* sp. commonly occurs above 25 °C in freshwater ecosystems [63,64]. It is possible that temperatures between 25–30 °C were optimal for emulating natural cyanobacterial blooms. Second, the cell scattering patterns of *Anabaena* sp. and *Microcystis* sp. partially overlapped with the scattering coordination because of their cell shape and size. Owing to these scattering patterns, *Anabaena* and *Microcystis* cells could not be perfectly distinguished because the forming patterns of the two live cells of cyanobacteria were calculated together rather than separately. This problem should be addressed in further studies by developing a distinguishing method for different cyanobacterial cell types.

Finally, our interpretations are based on metabolic flow and interactions between cyanobacteria and their associated bacteria. Although the experimental evidence for biochemical processes, especially nitrogen assimilation by cyanobacterial-associated bacteria, was not demonstrated in this study, the environmental and the water quality parameters as well as the metagenomic data of the bacterial composition revealed the possibility of relationships in a cyanobacterial phycosphere. Thus, symbiotic relationships and resource or nutrient exchanges between cyanobacteria and their associated bacteria under low light intensity conditions should be further investigated in future studies.

## 5. Conclusions

We investigated the effects of light on the growth of cyanobacteria (*Anabaena* and *Microcystis*) and the changes in bacterial communities in the cyanobacterial phycosphere using a large-scale cultivation system. The cell growth capacity and the growth patterns of *Anabaena* sp. and *Microcystis* sp., the two kinds of harmful cyanobacteria studied, differed under lightlimited conditions. The environmental and the water quality parameters indicated that nitrogen assimilation for the growth of cyanobacteria can occur primarily by nitrogen reductase under natural light conditions, whereas the alternative pathway (i.e., nitrogenase) in their associated bacteria can be activated under the blue light conditions. Cyanobacteria were found to have a symbiotic relationship with specific bacteria (e.g., *Sphingobium* sp.) through nitrogenous metabolites under the blue light conditions, which aided the growth of cyanobacteria. Green light conditions did not support the effective growth of cyanobacteria, even though comparable level of light intensity was provided in long wavelength range.

## Figures and Tables

**Table 1:** The variation in water quality in cyanobacterial culture in each experimental group (Control: No intervention, Blue: blue film, Green: green film, Blue/Green: blue and green film).

Factors	Conditions	0 h	24 h	72 h	144 h	196 h	240 h	312 h	360 h
BOD (mg/L)	Control	0.3	0.4	2.8	3.2	5.6	6.8	6.4	6.4
	Blue	0.3	0.8	0.8	1.6	2.8	2.4	3.2	0.2
	Green	0.3	0.8	3.6	2.4	2.4	2.4	4.4	0.2
	Blue/Green	0.3	0.4	4.0	2.0	2.0	3.6	3.6	0.4
COD (mg/L)	Control	1.0	1.6	4.4	6.0	8.8	6.8	9.2	7.2
	Blue	1.0	2.8	2.4	5.6	3.6	3.6	4.4	5.6
	Green	1.0	3.2	4.8	5.6	3.6	3.2	6.0	6.0
	Blue/Green	1.0	3.6	1.6	5.2	2.4	4.8	5.6	5.6
TN (mg/L)	Control	50.00	49.18	38.70	34.47	52.59	41.00	38.99	28.34
	Blue	50.00	46.67	54.30	38.32	47.55	48.87	54.42	35.28
	Green	50.00	55.66	57.82	37.59	42.37	48.51	57.94	43.12
	Blue/Green	50.00	49.50	37.98	39.98	37.74	38.55	38.24	37.94
TP (mg/L)	Control	1.00	0.98	0.75	0.60	0.87	0.63	0.74	0.53
	Blue	1.00	1.00	0.96	0.67	0.74	0.78	0.96	0.76
	Green	1.00	1.18	1.13	0.65	0.69	1.01	1.12	1.00
	Blue/Green	1.00	1.05	0.68	0.72	0.48	0.60	0.70	1.14
NO3-N (mg/L)	Control	52.85	55.57	52.44	21.33	27.64	20.48	23.97	30.08
	Blue	52.85	55.70	47.26	35.77	21.71	19.24	22.33	36.29
	Green	52.85	57.35	38.62	30.97	29.12	27.23	18.97	28.09
	Blue/Green	52.85	53.51	50.38	30.97	25.31	18.42	28.53	25.00
NO2-N (mg/L)	Control	0.02	0.02	0.05	0.14	0.27	0.18	0.18	0.23
	Blue	0.02	0.00	0.03	0.06	0.04	0.04	0.06	0.12
	Green	0.02	0.01	0.04	0.03	0.04	0.04	0.04	0.04
	Blue/Green	0.02	0.00	0.05	0.14	0.11	0.07	0.15	0.12
NH3-N (mg/L)	Control	0.08	0.00	0.00	0.00	0.04	0.07	0.18	0.14
	Blue	0.08	0.00	0.00	0.00	0.39	0.10	0.22	0.35
	Green	0.08	0.00	0.00	0.04	0.06	0.15	0.14	0.15
	Blue/Green	0.08	0.00	0.00	0.01	0.19	0.15	0.26	0.15
PO4-P (mg/L)	Control	1.01	0.93	0.69	0.34	0.37	0.38	0.38	0.39
	Blue	1.01	0.98	0.67	0.42	0.37	0.29	0.38	0.58
	Green	1.01	1.10	0.60	0.55	0.55	0.38	0.26	0.34
	Blue/Green	1.01	1.11	0.74	0.56	0.54	0.29	0.56	0.48

**Table 2:** Indicator species (or taxa) of bacterial community for five groups from cluster analysis.

Group	Indicator Species	IndVal	Freq	*p*
A	*Nordella*	1.00	4	0.001
	*Rhodobacter*	0.97	7	0.001
	*Aestuariisphingobium*	0.94	11	0.001
	*Novosphingobium*	0.86	28	0.002
	*Lentimicrobium*	0.75	3	0.004
	*Sphingorhabdus*	0.75	3	0.011
	*Herbaspirillum*	0.74	4	0.013
	*Geothrix*	0.50	2	0.043
	*Pelomonas*	0.49	4	0.047
	*Neochlamydia*	0.48	3	0.041
	*Tsukamurella*	0.46	3	0.047
B	*Sphingomonas*	0.91	28	0.002
	*Azohydromonas*	0.72	13	0.023
	*Simkania*	0.63	5	0.019
	*Reyranella*	0.60	24	0.008
	*Mucilaginibacter*	0.58	6	0.034
	*Gordonia*	0.57	20	0.003
	*Zoogloea*	0.54	23	0.017
C	*Sphingobium*	0.90	28	0.003
	*Flavobacterium*	0.87	23	0.006
	*Spirosoma*	0.85	14	0.003
D	*Bryobacter*	0.97	6	0.001
	*Roseomonas*	0.97	9	0.001
	*Lentilitoribacter*	0.95	8	0.001
	*Hymenobacter*	0.92	7	0.001
	*Blastomonas*	0.92	10	0.001
	*Peredibacter*	0.75	6	0.032
	*Fimbriimonas*	0.75	4	0.012
	*Gemmobacter*	0.72	27	0.001
	*Bosea*	0.64	28	0.005
	*Devosia*	0.60	28	0.001
	*Sphingopyxis*	0.59	20	0.004
	*Rhizobium*	0.59	28	0.001
	*Chryseolinea*	0.57	24	0.022
	*Phreatobacter*	0.56	13	0.018
	*Hydrogenophaga*	0.55	28	0.018
	*Ensifer*	0.54	28	0.002
	*Sediminibacterium*	0.52	28	0.001
	*Exiguobacterium*	0.50	2	0.038
	*Sediminicoccus*	0.50	2	0.031
	*Simplicispira*	0.50	2	0.027
E	*Cupriavidus*	0.95	23	0.010
	*Porphyrobacter*	0.93	15	0.001
	*Bradyrhizobium*	0.78	28	0.001
	*Cylindrospermum*	0.74	28	0.001
	*Brevundimonas*	0.70	27	0.001
	*Limnobacter*	0.65	15	0.001
	*Caulobacter*	0.63	27	0.006
	*Ralstonia*	0.61	26	0.020
	*Methylobacterium*	0.57	28	0.003
	*Aeromicrobium*	0.50	5	0.041
	*Acidovorax*	0.38	28	0.039

## Data Availability

The meta-amplicon sequencing datasets generated for this study can be found in the Sequence Read Archive of NCBI. Project number PRJNA740357 is available at https://www.ncbi.nlm.nih.gov/sra/?term=PRJNA740357 (accessed on 18 September 2022). All other data generated or analyzed during this study are included in this article and its Supplementary Data.

## Supplementary Materials

The following supporting information can be downloaded at: https://www.mdpi.com/article/10.3390/microorganisms10112150/s1, Figure S1: Quantification for cyanobacterial cells using Guava flow cytometry; Figure S2: Result of flow cytometry analysis profile during the cyanobacterial cultivation period; Figure S3: Rarefaction curve for the meta-amplicon sequencing of four experimental groups; Table S1: The variation in physico-chemical factors in cyanobacterial culture in each experimental group; Table S2: Alpha diversity index of four experimental groups.
