# Supplementary material for: Comparison of Bacterial Assemblages Associated with Harmful Cyanobacteria under Different Light Conditions
Source: Microorganisms. 2022 Oct 30;10(11):2150. doi: 10.3390/microorganisms10112150 (PMC9694310; doi:10.3390/microorganisms10112150)
Supplement: Supplementary file 1 [file microorganisms-10-02150-s001.zip › microorganisms-1951611-supplementary.pdf]

Supplementary Materials

Article

Comparison of Bacterial Assemblages Associated with Harmful Cyanobacteria under Different Light Conditions

Taehui Yang [1,†], Chang Soo Lee [2,†], Ja-Young Cho [2], Mi-Jung Bae [2,*] and Eui-Jin Kim [2,*]

[1] Gossong Deep Sea Water Industry Foundation, Gossong-gun 24747, Korea
[2] Nakdonggang National Institute of Biological Resource (NNIBR), Sangju-si 37242, Korea
* Correspondence: mjbae@nnibr.re.kr (M.-J.B.); ejkim@nnibr.re.kr (E.-J.K.)
† These authors contributed equally to this work.

Figure S1. Quantification for cyanobacterial cells using Guava flow cytometry (X and Y axis are arbitrary units). (a) *Microcystis* sp. cells (b) *Anabaena* sp. cells. (First square: forward scatter and side scatter, second square: forward scatter and red-B fluorescence and third square: forward scatter and green fluorescence).

Figure S2. Result of flow cytometry analysis profile during the cyanobacterial cultivation period (X and Y axis are arbitrary units; first square: forward scatter and side scatter, second square: forward scatter and red-B fluorescence and third square: forward scatter and green fluorescence). (a), (b), (c), (d), (e), (f), (g) and (h): cultivation day 1 to day 15.

Figure S3. Rarefaction curve for the meta-amplicon sequencing of four experimental groups.

Table S1. The variation in physico-chemical factors in cyanobacterial culture in each experimental group.

Table S2. Alpha diversity index of four experimental groups. (a) Chao1 index, (b) Shannon index.

Figure S1. Quantification for cyanobacterial cells using Guava flow cytometry (X and Y axis are arbitrary units). (a) *Microcystis* sp. cells (b*)* *Anabaena* sp. cells. (First square: forward scatter and side scatter, second square: forward scatter and red-B fluorescence and third square: forward scatter and green fluorescence).

(a)

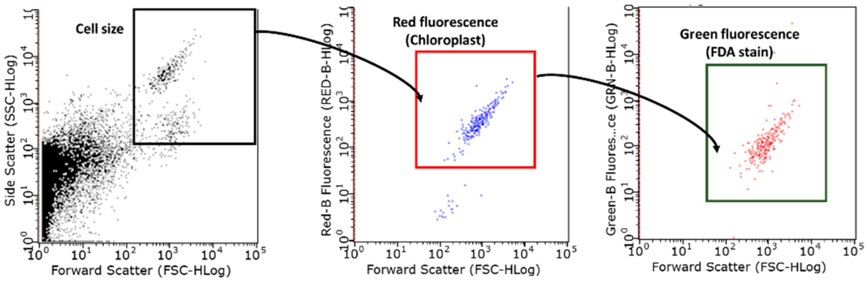

(b)

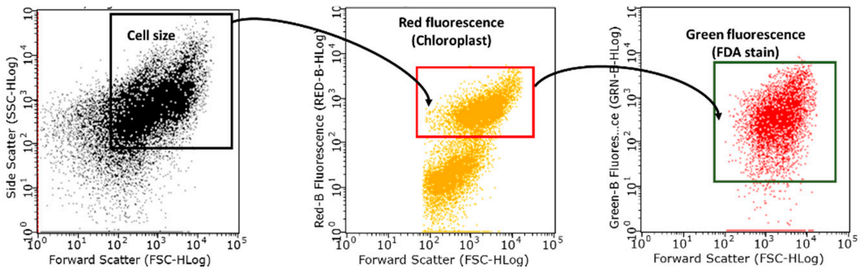

Figure S2. Result of flow cytometry analysis profile during the cyanobacterial cultivation period (X and Y axis are arbitrary units; first square: forward scatter and side scatter, second square: forward scatter and red-B fluorescence and third square: forward scatter and green fluorescence). (a), (b), (c), (d), (e), (f), (g) and (h): cultivation day 1 to day 15.

(a)

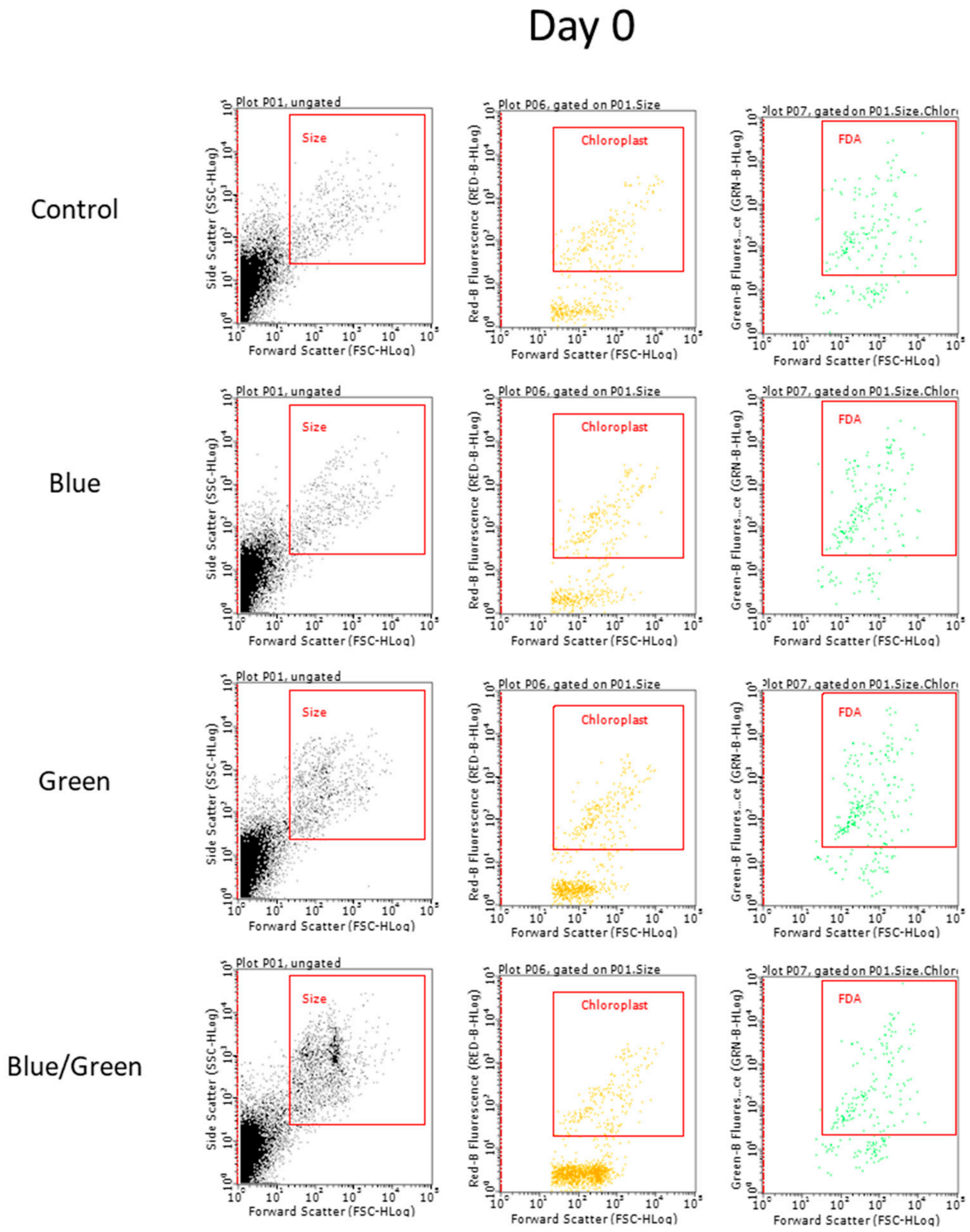

(b)

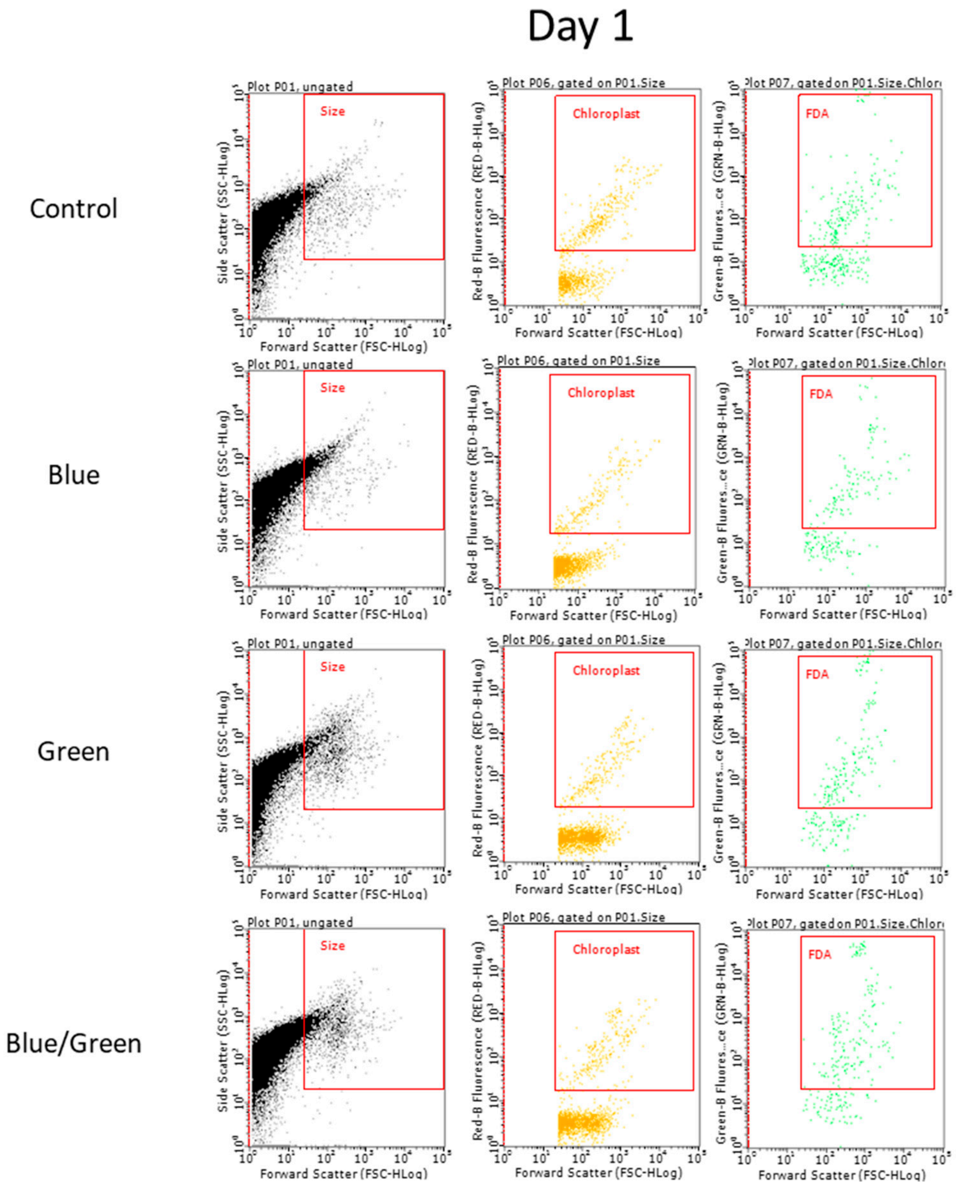
Day 1
Control
Blue
Green
Blue/Green
Plot P01, ungated
Size
Side Scatter (SSC-HLog)
Forward Scatter (FSC-HLog)
Plot P06, gated on P01.Size
Chloroplast
Red-B Fluorescence (RED-B-HLog)
Plot P07, gated on P01.Size.Chlor
FDA
Green-B Fluores...ce (GRN-B-HLog)

(c)

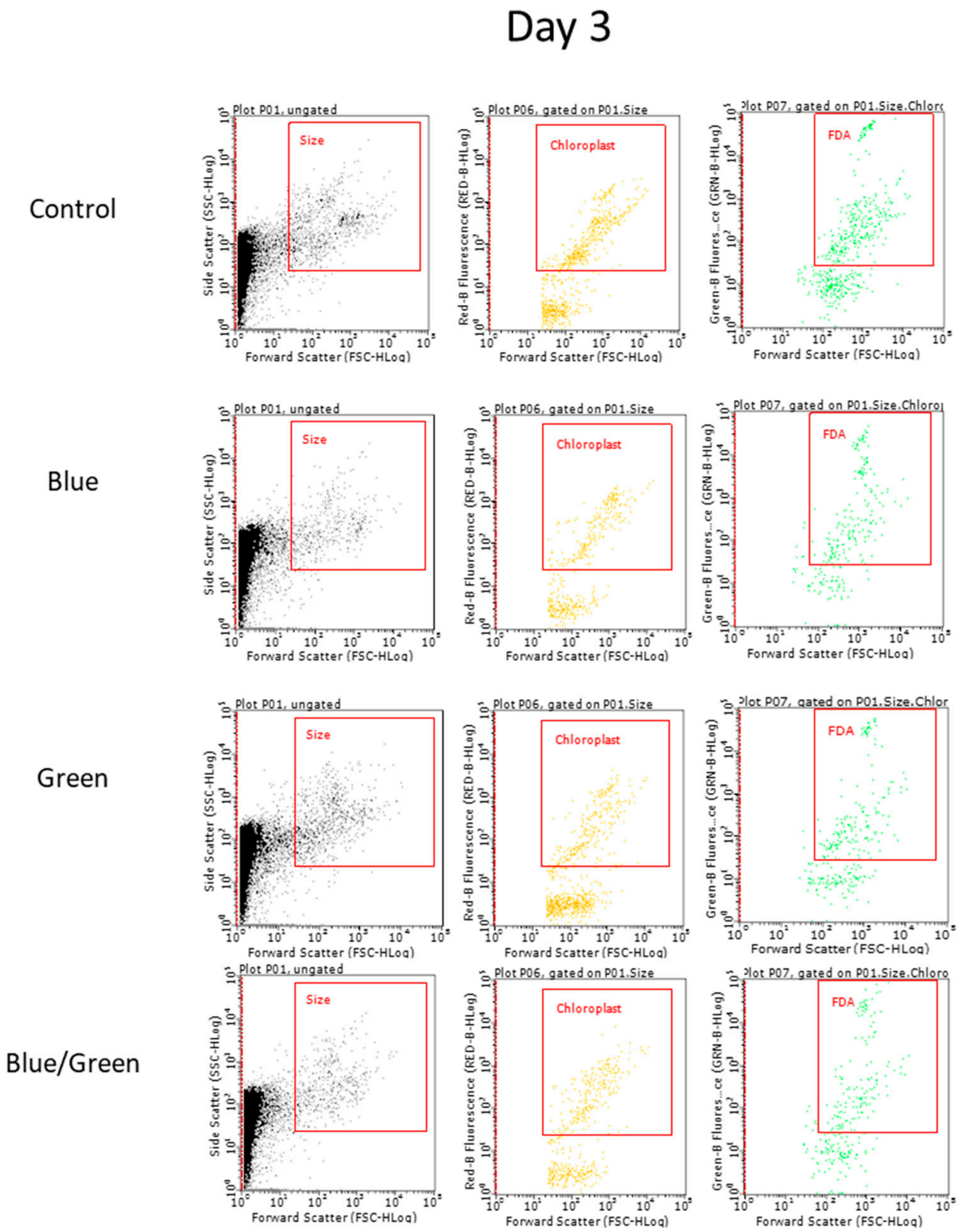
Day 3
Control
Blue
Green
Blue/Green
Plot P01, ungated
Plot P06, gated on P01.Size
Size
Chloroplast
FDA
Side Scatter (SSC-HLog)
Red-B Fluorescence (RED-B-HLog)
Forward Scatter (FSC-HLog)

(d)

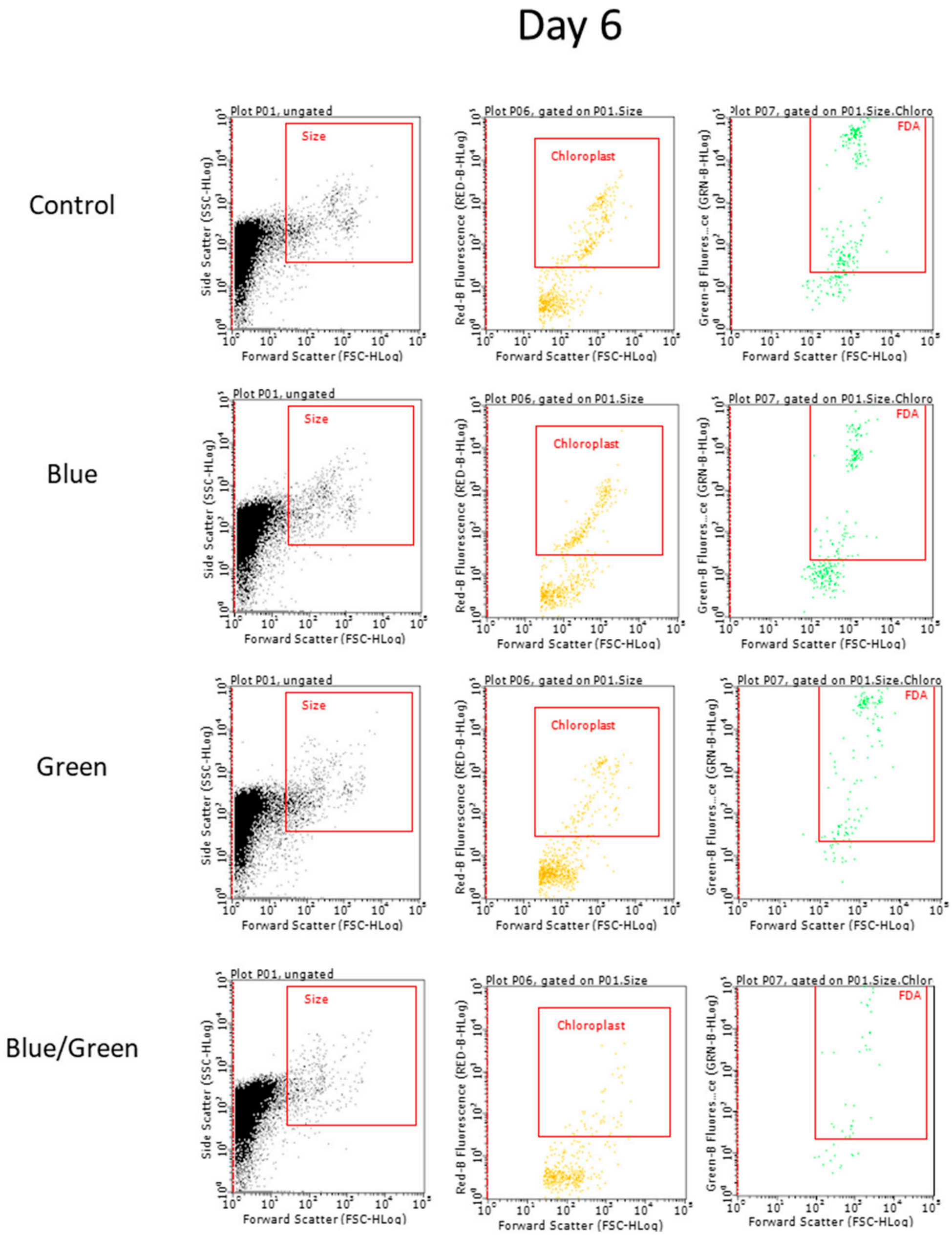

Day 6
Control
Blue
Green
Blue/Green
Plot P01, ungated
Plot P06, gated on P01.Size
Plot P07, gated on P01.Size.Chloro
Size
Chloroplast
FDA
Side Scatter (SSC-HLog)
Red-B Fluorescence (RED-B-HLog)
Green-B Fluores...ce (GRN-B-HLog)
Forward Scatter (FSC-HLog)

(e)

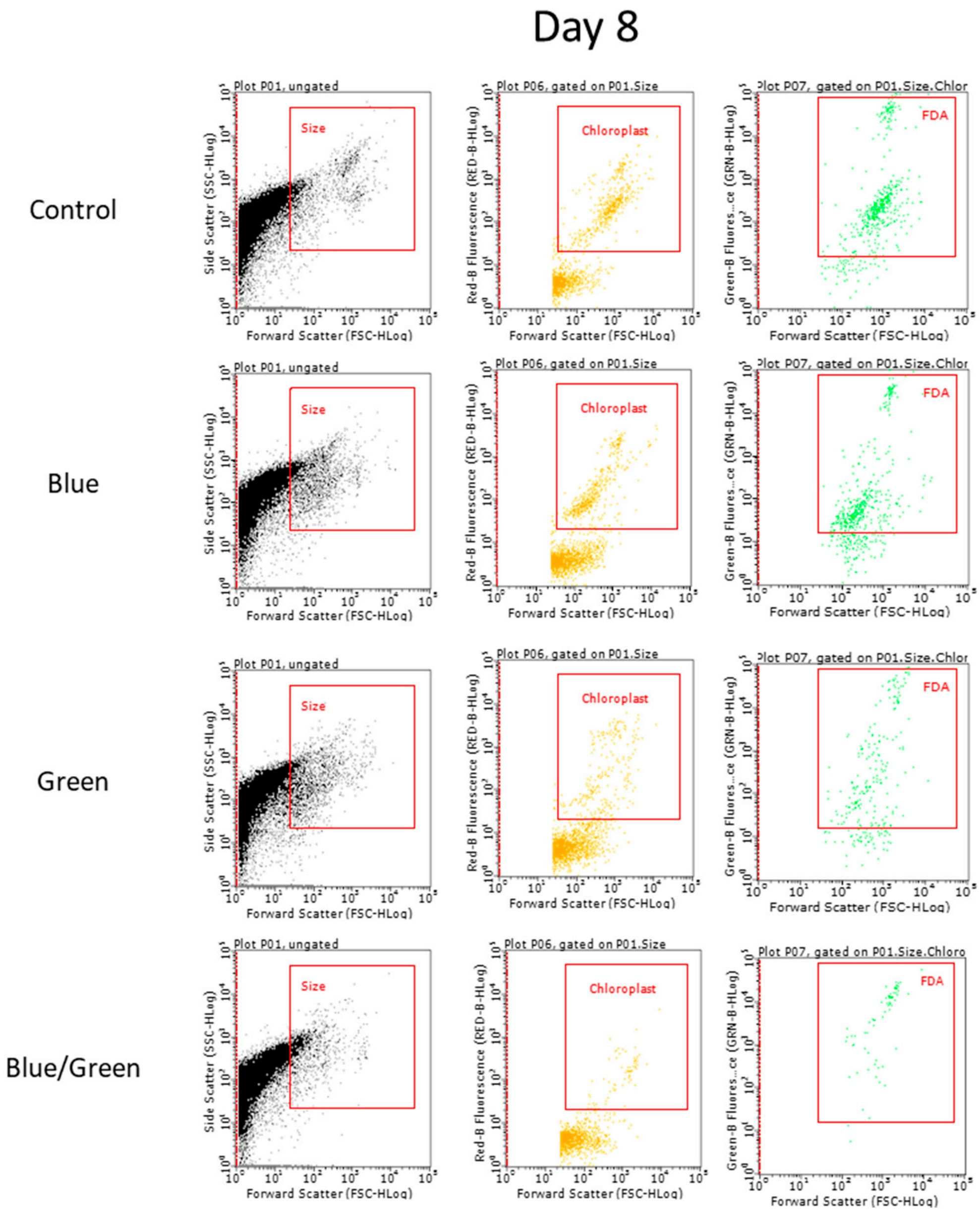
Day 8
Control
Blue
Green
Blue/Green
Plot P01, ungated
Size
Side Scatter (SSC-HLog)
Forward Scatter (FSC-HLog)
Plot P06, gated on P01.Size
Chloroplast
Red-B Fluorescence (RED-B-HLog)
Plot P07, gated on P01.Size.Chlor
FDA
Green-B Fluores...ce (GRN-B-HLog)

(f)

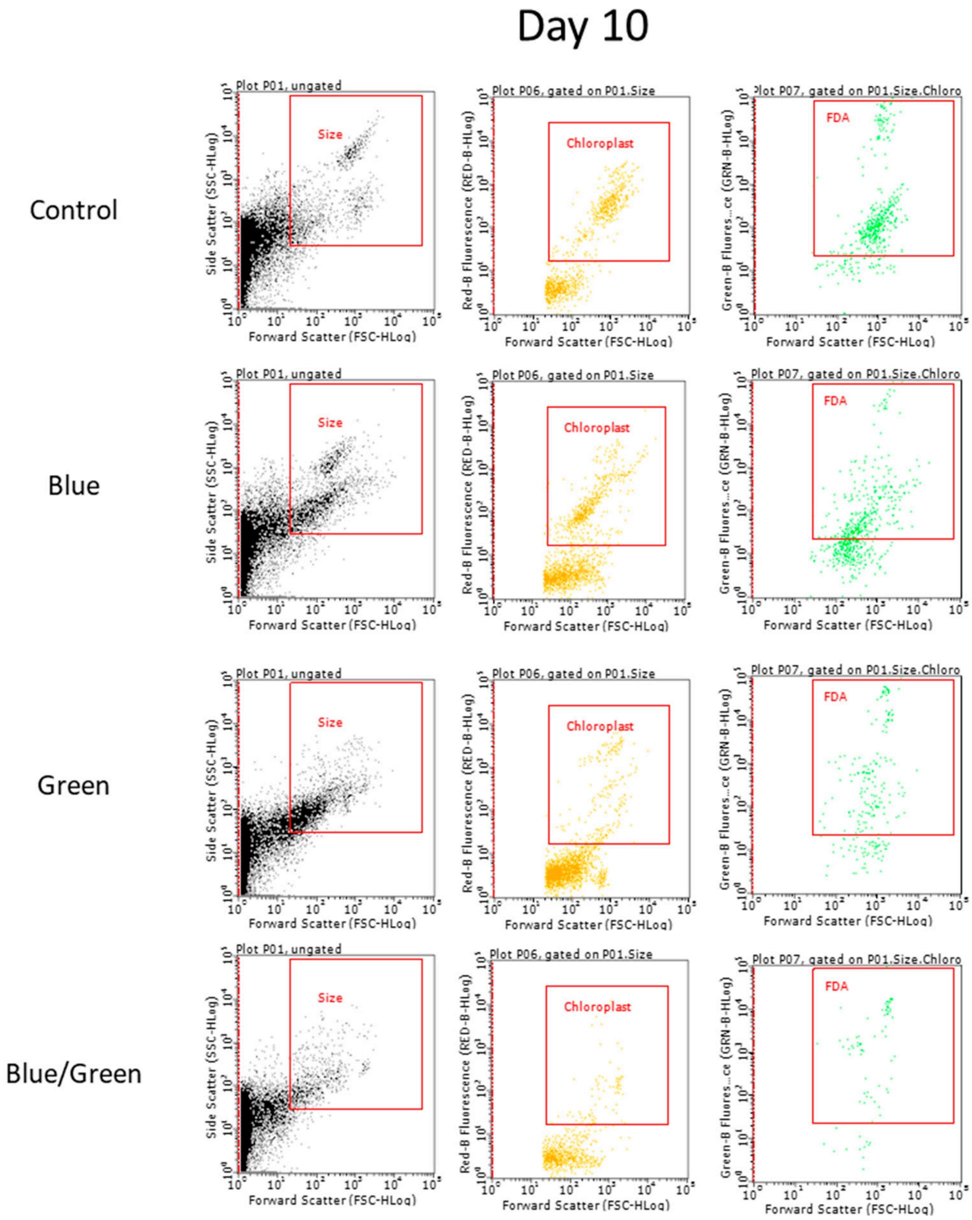
Day 10
Control
Blue
Green
Blue/Green
Plot P01, ungated
Plot P06, gated on P01.Size
Plot P07, gated on P01.Size.Chloro
Size
Chloroplast
FDA
Side Scatter (SSC-HLog)
Red-B Fluorescence (RED-B-HLog)
Green-B Fluores...ce (GRN-B-HLog)
Forward Scatter (FSC-HLog)

(g)

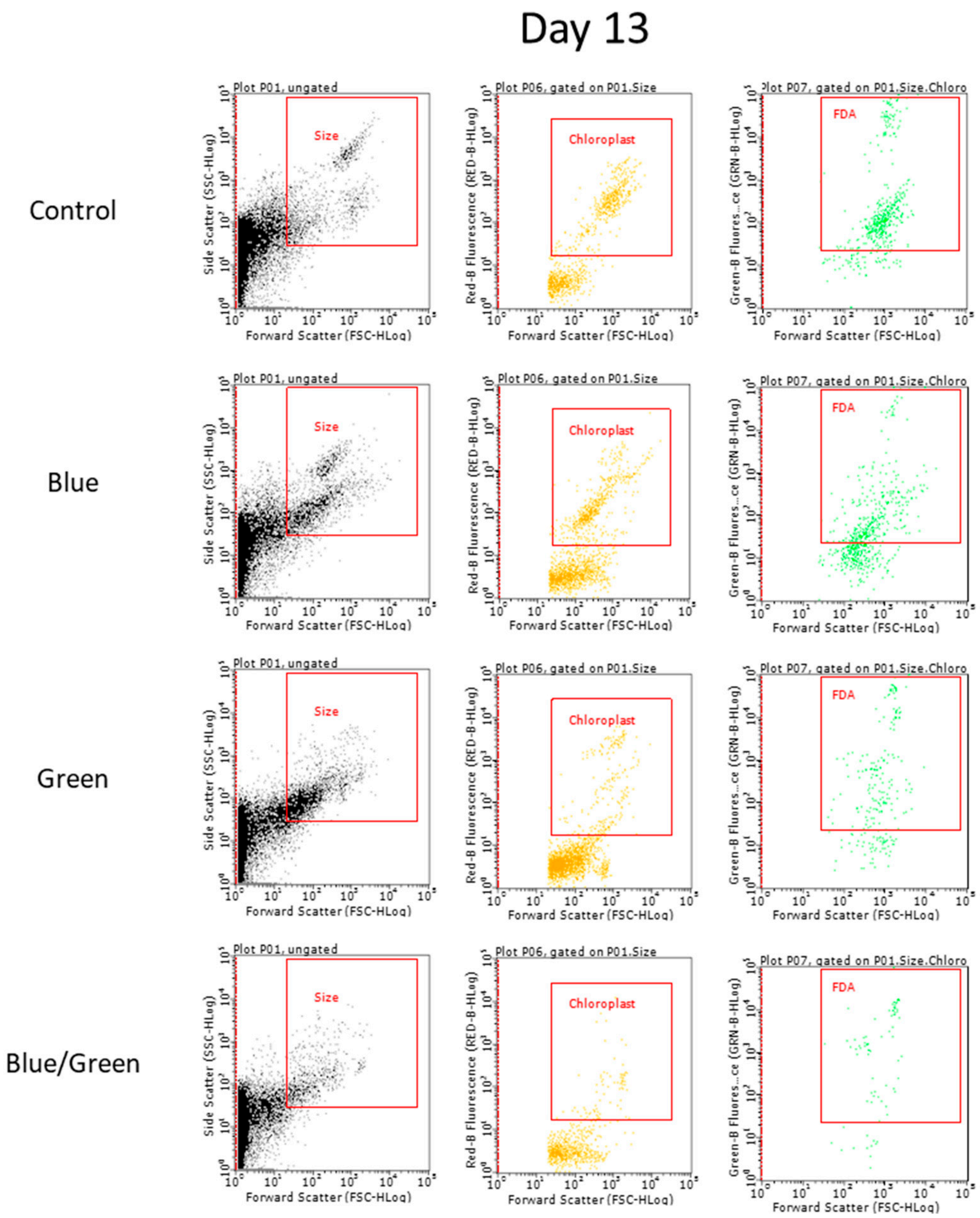

Day 13
Control
Blue
Green
Blue/Green
Plot P01, ungated
Plot P06, gated on P01.Size
Plot P07, gated on P01.Size.Chloro
Size
Chloroplast
FDA
Side Scatter (SSC-HLog)
Red-B Fluorescence (RED-B-HLog)
Green-B Fluores...ce (GRN-B-HLog)
Forward Scatter (FSC-HLog)

(h)

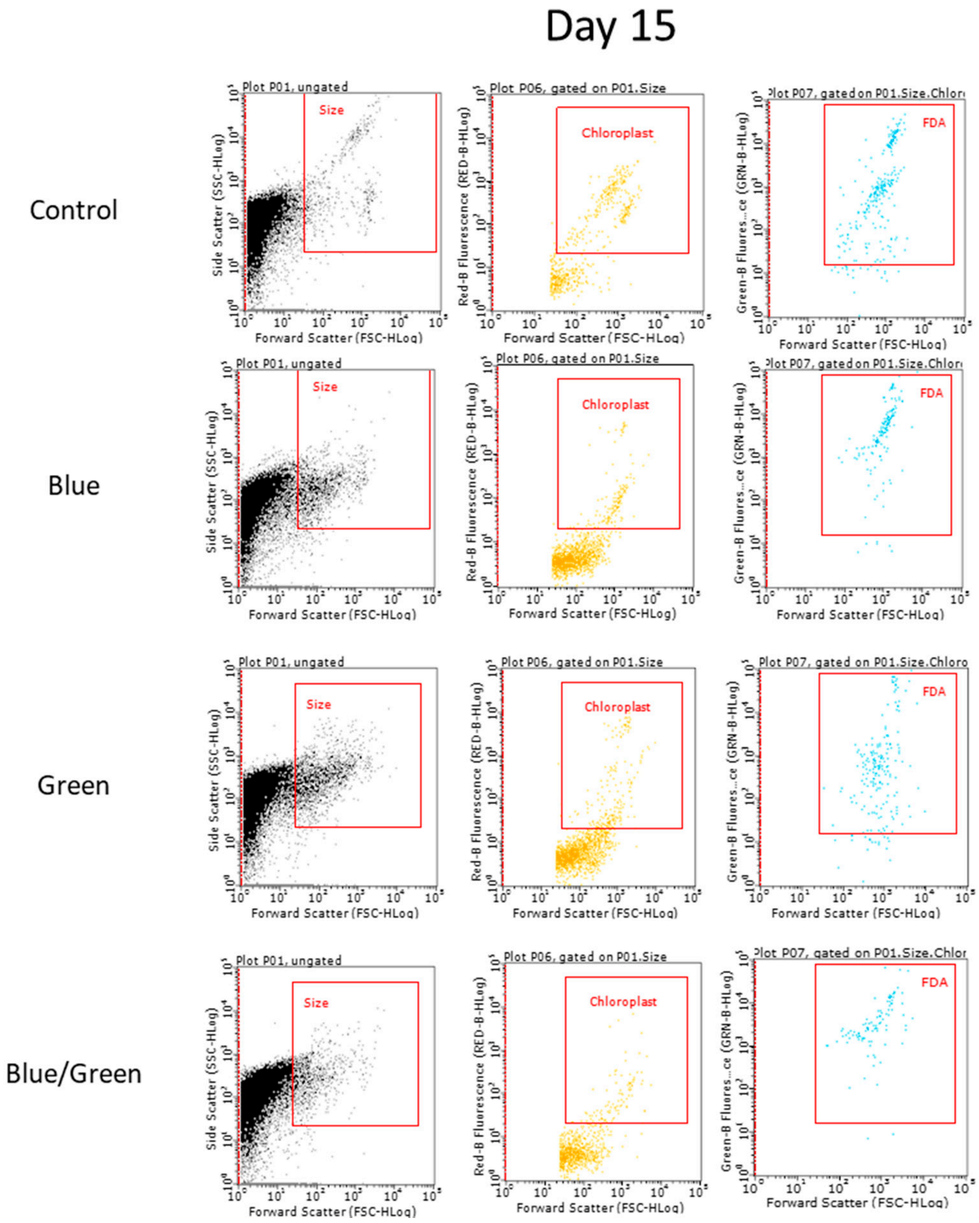
Day 15
Control
Blue
Green
Blue/Green
Plot P01, ungated
Plot P06, gated on P01.Size
Size
Chloroplast
FDA
Side Scatter (SSC-HLog)
Red-B Fluorescence (RED-B-HLog)
Green-B Fluores...ce (GRN-B-HLog)
Forward Scatter (FSC-HLog)

Figure S3. Rarefaction curve for the meta-amplicon sequencing of four experimental groups.

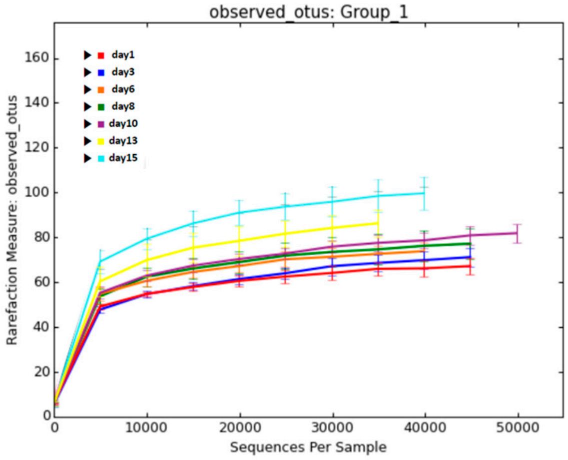

Table S1. The variation in physico-chemical factors in cyanobacterial culture in each experimental group.

Factors	Conditions	0 h	24 h	72 h	144 h	192 h	240 h	312 h	360 h
Temperature (°C)	Control	24.1	25.1	27.6	29.5	30	27.1	27.3	27.3
	Blue	24.1	25.1	27.6	29.7	30.3	27.6	27.7	27
	Green	24.1	25.1	27.6	29.7	30	27.6	27.7	27.7
	Blue/Green	24.1	24.9	27.4	29.7	30.1	27.6	27.6	27.1
DO (%)	Control	110.1	108.4	127.3	120	102.7	100.4	94.4	100
	Blue	110.1	108.4	115.2	112	105.3	101.4	99.6	96.5
	Green	110.1	102.6	107.7	105	101.4	100.3	86.1	85.1
	Blue/Green	110.1	100.6	103.1	101	100.9	99.9	100.3	95.1
pH	Control	8	8.12	10.4	8.69	9.58	8.09	9.31	9.9
	Blue	8	8.12	9.2	8.03	7.72	7.82	7.55	7.88
	Green	8	7.32	8.67	7.85	7.83	7.65	7.86	7.75
	Blue/Green	8	7.19	7.66	7.48	7.36	7.38	7.4	7.28
Turbidity (FNU)	Control	2.5	2.6	3.22	2.4	6.78	9.53	18.02	22
	Blue	2.5	2.6	2.82	2.8	4.82	4.57	2.71	2.7
	Green	2.5	2.41	3.23	2.87	3.86	3.41	2.74	2.69
	Blue/Green	2.5	0.14	0.5	1.08	0.35	0.36	0.02	0.00

Table S2. Alpha diversity index of four experimental groups.

Sample name		OTUs	Chao1	Shannon
Control group	Day 1	74	78.20	3.61
	Day 3	73	95.00	3.03
	Day 6	70	70.14	3.37
	Day 8	88	90.00	3.43
	Day 10	75	82.00	3.14
	Day 13	98	109.00	4.08
	Day 15	93	98.00	3.80
Blue group	Day 1	67	70.00	3.66
	Day 3	78	93.00	4.11
	Day 6	83	96.20	4.47
	Day 8	69	69.50	2.70
	Day 10	83	98.60	3.28
	Day 13	81	90.17	2.39
	Day 15	115	125.46	2.63
Green group	Day 1	70	81.25	3.11
	Day 3	74	75.00	2.51
	Day 6	73	73.60	3.66
	Day 8	75	78.33	3.72
	Day 10	85	90.14	3.97
	Day 13	92	108.50	4.11
	Day 15	107	115.25	4.30
Blue/Green group	Day 1	65	75.50	3.36
	Day 3	67	85.33	3.50
	Day 6	76	80.00	3.77
	Day 8	80	101.00	3.34
	Day 10	87	133.20	3.38
	Day 13	84	91.09	2.90
	Day 15	94	105.14	3.20
